# Strengthening complex systems for chronic disease prevention: a systematic review

**DOI:** 10.1186/s12889-019-7021-9

**Published:** 2019-06-11

**Authors:** Lori Baugh Littlejohns, Andrew Wilson

**Affiliations:** Menzies Centre for Health Policy, The Australian Prevention Partnership Centre, D17 Charles Perkins Centre, University of Sydney, Sydney, NSW 2006 Australia

**Keywords:** Chronic disease, Prevention, Complex systems, Framework

## Abstract

**Background:**

While frameworks exist for strengthening health care systems and public health systems, there are no practical frameworks to describe, assess and strengthen systems for chronic disease prevention (CDP) using complex systems approaches.

**Methods:**

A systematic and integrative review of peer reviewed literature was conducted to answer the following questions: How can systems for CDP be defined? What are key attributes of effective systems? How are complex systems approaches discussed? Search terms were identified and the Medline, SCOPUS, and Global Health databases were searched December 2017 and January 2018. Reference lists and selected journals were hand searched. A working definition for a system for CDP was developed to provide a guideline for inclusion. Key exclusion criteria were literature did not address the research questions or working definition; was published in a language other than English and before 2000; focused on specific chronic diseases and/or risk factors and not CDP broadly; concentrated on the health care sector and clinical services and/or health status and surveillance data; and described evaluations of setting specific actions such as policies, programs, interventions, approaches, projects, laws, or regulations. Selected literature (*n* = 141) was coded in terms of the extent to which the research questions and the working definition of systems for CDP were addressed. Data was then analysed and synthesized to determine key themes.

**Results:**

A revised definition of systems for CDP and seven attributes of effective systems for CDP are reported (collaborative capacity, health equity paradigm, leadership and governance, resources, implementation of desired actions, information and complex systems paradigm). A framework was developed to provide a foundation for describing, assessing and strengthening systems for CDP.

**Conclusions:**

The results of this literature review provide a strong foundation for a framework to help strengthen systems for CDP. The framework consolidates not only well-established attributes of effective CDP but also highlights theoretical and practical insights from complex systems perspectives.

**Electronic supplementary material:**

The online version of this article (10.1186/s12889-019-7021-9) contains supplementary material, which is available to authorized users.

## Background

German scientist Georg Lichtenberg is famous for stating *I cannot say whether things will get better if we change; what I can say is they must change if they are to get better.* This is an apt expression of the urgency to address the persistently high rates of chronic disease around the world [1]. The study and application of new complex systems approaches to chronic disease prevention (CDP) is increasingly called for to address this intractable problem [2–4]. Wutzke et al. [5] report that complex systems approaches are needed to affect change in the dynamic relationships among people, entities, processes, activities, settings and structures that facilitate or hinder CDP efforts. In essence, systems must change and be strengthened if CDP is to get better.

Compelling frameworks exist for strengthening *health care systems* and *public health systems* but they do not exist for complex systems for CDP. For example, the World Health Organization [6] provides a framework for strengthening health systems that consists of six building blocks that includes leadership, resources, service delivery, workforce, information, and medicines and technology. However, health systems are defined in terms of entities and activities with the *primary* mandate for health whereas entities and activities required in CDP most often lie outside the health system. Therefore, they may only have health as an implicit or tangential outcome (e.g., active transportation) if at all. Systems for CDP must include a multitude of sectors without a primary mandate for health. Furthermore, frameworks to strengthen public health systems also exist [7–11]. However, these frameworks apply very broad lenses to describe public health structures (e.g., workforce, financing), functions (e.g., service delivery) and classifications (e.g., settings) but do not provide the detail necessary to adequately describe the multisectoral nature of systems for CDP. Although we found some frameworks that focus on CDP [12–19] these do not define systems for CDP, describe attributes of effective systems in a comprehensive manner, or offer practical recommendations for assessing and strengthening the diversity of systems for CDP using complex systems approaches.

Thus, there is a need to develop a framework to not only help communicate what is meant by a system for CDP but to also provide recommendations for practical assessment approaches to identify key leverage points to strengthen systems to reduce rates of chronic disease. The aim of this paper is to report on the results of a literature review that provide the foundation for a practical framework to strengthen systems for CDP and answer the following research questions: How can systems for CDP be defined? What are key attributes of effective systems for CDP? How are complex systems approaches discussed with respect to systems for CDP?

## Methods

A systematic and integrative literature review was conducted and this approach was chosen because knowledge gained is “synthesized into a model or conceptual framework that offers a new perspective on the topic” [20]. The search strategy was based upon recommended methods for this type of review [21], examples in the literature [22], and the advice of a university librarian. Key search terms are listed in Table 1 and the Medline, SCOPUS, and Global Health databases were searched December 2017 and January 2018. Reference lists and selected journals were hand searched to identify literature not found in the database searches and had the potential to contribute to the focus on systems for CDP.

**Table 1:** Search terms

chronic disease*, NCD*, non?communicable, prevent*, reduc*, system*, capacity*, public health, “population health”, approach*, barrier*, challenge*, strateg*, politic*, whole-of-society, whole-of-gov, “health in all polic*”, determinant*, society, healthy public policy, multi*, multisector*, multidisciplin*, intersector*, partner*, collab*, coordinat*, stakeholder*, infrastructure, network*, organi?ation*, multi-level, workforce*, sector*, resource*, ecolog*, adaptive system, system* thinking, complexity.	

Grey literature was searched during a prior proof-of-concept study undertaken to review Australian (national and state/territory) and international strategic documents relevant to systems for CDP. The health system building block framework described above was used to study these documents and although we found it useful we questioned if other attributes might ascend in importance in an effective system for CDP (as opposed to a health care system). Thus, the impetus for this literature review.

We found no definitions of a system for CDP, therefore, a working definition was developed to provide a guideline for inclusion. The work of de Savigny and Adam [23], Fawcett et al. [16], Contandiopoulos et al. [24] and the NICHSR [9] were helpful and the working definition used was *multiple entities and actions that work in dynamic ways to affect the complex array of factors that contribute to CDP*. ‘Actions’ were collectively taken to include policies, programs, interventions, strategies, approaches, projects, laws, and regulations [25].

Key exclusion criteria were that literature: a) did not address the research questions or working definition, b) was published in a language other than English and before 2000, c) focused on a specific chronic disease and/or risk factors and not CDP broadly, d) concentrated on the health care sector, clinical services and/or health status and surveillance data, and e) described evaluations of setting specific actions. If the literature was considered borderline with respect to the above criteria, it was generally included.

**Fig. 1:**
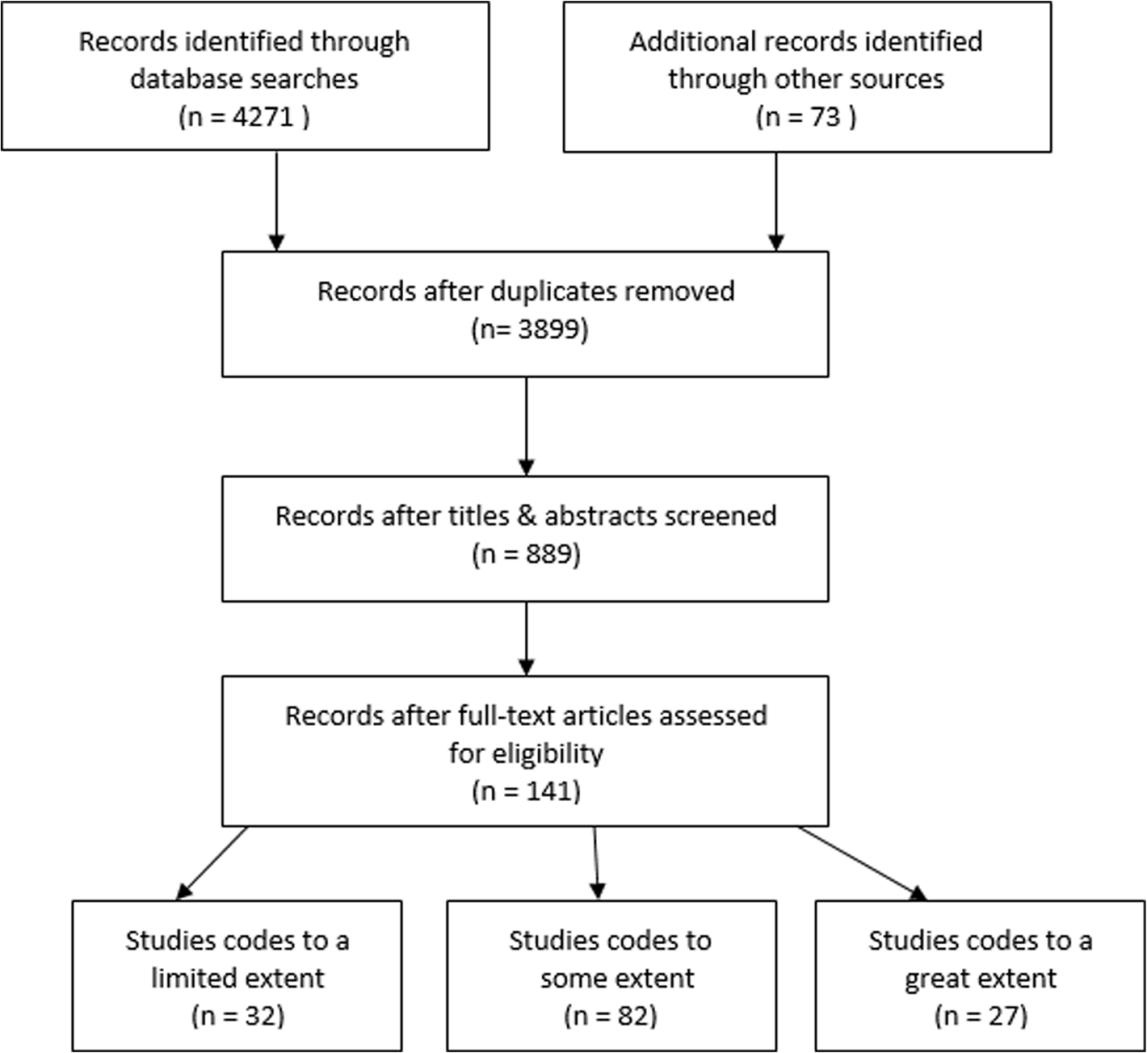
Prisma diagram

A total of 4271 records from database searches and 73 records from hand searches were identified (Fig. 1). After removal of duplicates and a review of titles and abstracts, a total of 444 records were downloaded to an EndNote database. A full text review of articles was completed and in total, 141 records were included for review. The selected records broadly reflected key literature based upon our search strategy and were not considered to be an exhaustive list.

To support data extraction and analysis, selected literature was assessed and coded according to qualitative directed content processes, that is, through the identification of pre-set categories [26]. Using an Excel spreadsheet three categories were first established: *limited extent* (discussed multiple entities and actions but did not explicitly address attributes of systems for CDP nor dynamic interactions), *some extent* (discussed multiple entities and actions and attributes of effective systems for CDP but did not discuss dynamic interactions), and *great extent* (discussed all of the above). Of the 141 records, 32 were coded to a *limited extent*, 82 to *some extent* and the remaining 27 to a *great extent (*Additional file 1). To test reliability of coding, 25 records were coded by two reviewers (first author and research assistant) and differences were discussed and consensus was reached. The remaining records (*n* = 117) were divided between the two reviewers for coding. Data was then extracted in terms of descriptions of a) diverse entities or parts of the system, b) attributes of effective systems, and c) complex systems approaches. Following this, descriptions were analysed to identify patterns (first author) and key themes were labelled based upon these patterns.

## Results

This section is organized into two parts. The first section reports on themes with respect to how diverse entities or parts of the system are described and the second describes seven themes or attributes of effective systems for CDP. Included in the latter is reporting on complex systems approaches.

### Systems for chronic disease prevention

Overall, systems for CDP were described in terms of diverse entities and multiple sectors that work at multiple levels to prevent chronic disease -- that particularly include governments, health systems, and topic focused entities -- in contexts that are unique and ever changing due to the dynamic nature of the relationships among entities and actions. Building on the working definition (indicated above), the literature was profuse in describing the breadth and scope of elements of systems for CDP and Table 2 provides a summary.

**Table 2:** Description of elements of systems for CDP

Key elements	Description
Diverse entities and multiple sectors	Governments and their departments (e.g., health, agriculture, transportation, trade, education, planning), civil society, philanthropic organisations, international agencies, private sector, and entities focused on research, policy, and practice [27–33].
Multiple levels	Global/international [34–39], federal, national, state/provincial or regional, and municipal or local [14, 40–50] through to community and individuals [51].
Unique and ever changing contexts	History, geography, values, social interactions and structures, political and legal systems, economic situations, existing resources, policies and initiatives, features of entities or organizations, and physical environments [46, 52, 53].
Dynamic relationships or interactions	Structures and processes that link people, entities and actions to prevent chronic disease [15, 19, 54, 55]

### Attributes of effective systems for chronic disease prevention

Seven attributes of effective systems for CDP were identified and these are described below: collaborative capacity, leadership, health equity paradigm, resources, implementation of desired action, information, and a complex systems paradigm.

#### Collaborative capacity

Collaborative capacity and having a collaborative mindset was discussed in all literature reviewed. Some was particularly strong in this regard [14, 19, 28, 37, 54, 56–75] while other literature made mention of the need for collaboration. Having a capacity building mindset was described as having a systematic approach to continually assess existing collective system capacity [76] and Manson et al. [14] argued that this mindset was especially needed among leaders in the public sector. There were plentiful and broad descriptions regarding critical success factors of multisectoral collaboration or partnerships [47–50, 54, 77, 78]. It is beyond the scope of this paper to elaborate in detail on success factors, however, the need for effective mechanisms, structures, processes, and facilitative infrastructure for coordination, collaboration, and relationship building were widely discussed [16, 19, 28, 74, 77, 79–82]. Woulfe et al. [77] maintained that collaborative partnerships were in essence the system for CDP.

Some literature focused on the unique challenges of building collaborative capacity in public – private partnerships for CDP [28, 45, 54, 83]. The need for rules of engagement, resolution of conflicts of interest and governance mechanisms to protect public health policies from industry interference were particularly discussed [31, 35, 39, 58, 64, 84].

Another theme regarding collaborative capacity was the need for active participation and collaboration with community members [55, 81, 85–89]. The term ‘community’ was often used to broadly describe the importance of collaborating with and empowering people at the local level [51, 75, 90]. Some literature was detailed in reporting that community-level governance is necessary [91] and that good governance requires “active citizenship and participation in deliberative democracy to form social consensus” [92]. This theme was reinforced through discussions of community capacity building, empowerment approaches and community-based participatory action research [38, 52, 93].

#### Leadership

A frequently discussed attribute of effective systems for CDP was not only leadership but linked dimensions of governance and accountability. In terms of leadership, there was considerable emphasis on the need for demonstrated political will to ensure CDP actions are embedded in policy agendas [14, 15, 94–96]. For example, Swinburn [97] stated that “the impetus for change needs a critical level of political leadership and some defined policy directions” (p4). The links to policy leadership for CDP were abundant [14, 34, 46, 51, 98, 99]. High-level leadership - people who can influence change at the policy level - was described as necessary to establish legitimacy [54] and priority or buy in for CDP [43, 100, 101]. Furthermore, the need for high-level support for action on the social determinants of health was a very common theme [19, 43, 68, 95, 102, 103]. Academic leadership [104, 105] and political champions or leaders in civil society [95] were also highlighted. However, it was leadership from within the health sector that was most emphasized [70, 96, 106–109].

Leadership at multiple levels was also identified. Although global [37, 94], state [82, 104] and local levels [40] were discussed, it was national- level leadership that was most frequently reported as important to effective systems for CDP [17, 44, 45, 54, 76, 97, 110, 111]. National leadership was considered critical to oversee and integrate strategic policy frameworks [5, 44, 74], align vision, strategy and mission [19, 83] in integrated plans and joined up governance structure [46, 51, 58, 65, 67, 69, 74, 76, 79, 112–116]. Leadership at many levels in many sectors was often reported as essential [98, 117], for example, public sector leadership at all jurisdictional levels was emphasized by Manson et al. [14] and Swinburn [97].

Regarding governance, the following elements were described as important to effective systems for CDP: transparent structures and processes, strategic frameworks, multisectoral coordination, coalitions and partnerships, policy development and planning [5, 19, 54, 69, 74]. Discussion of planning and the development of policy, regulations and laws were closely linked to the need for systems of integrated and multilevel governance [95, 99, 118], health in all policies [101, 115] and multisectoral public health policy [32, 42, 63, 80, 119–123]. Abernethy [75] specifically discussed the need for a paradigm shift to complex socio-ecological systems governance that “includes social justice, gender equity, inclusive participatory engagement and transparency in deliberative processes” (p457). The critical connections or relationships between collaborative capacity, national leadership for joined up governance, and a health equity paradigm (discussed below) were evident in the literature.

Finally, accountability was mostly discussed in terms of the critical leadership and governance role in system for CDP. For example, Gostin et al. [58] discussed multisectoral accountability in governance mechanisms and Beaglehole et al. [38] identified the need for multilevel (national and international) governance and accountability for CDP. The focus was largely on monitoring and evaluation of outcomes in CDP and this is discussed below as a dimension of the information attribute. Thus, more connections between attributes such as leadership and information emerged from the literature review.

#### Health equity paradigm

There was a strong theme that effective systems for CDP need a health equity paradigm as a foundation. This was discussed as relating to a social justice or human rights approach through action on the social determinants of health [14, 32, 39, 75, 90, 102, 114, 118, 124]. A human rights approach was described as requiring a paradigm shift focused on the structural determinants of chronic disease [14, 19, 34, 46, 75, 101, 118, 122, 125]. Shifting the paradigm was reported in such terms as increasing the value of health as a public good and of collective action [88, 126]. Some literature focused on action on the social determinants of health with little discussion of health equity [41, 44, 45]. Furthermore, there was little in the literature that explicitly described how this would be realized in practice (e.g., priority setting for health equity). As noted above, a health equity paradigm was linked to system leadership and governance in some literature.

#### Implementation of desired action

There was a theme that CDP suffers from a lack of effective implementation of desired action and that continued focus on planning is problematic [19, 25, 90, 93, 127, 128]. The reasons for the lack of implementation were not clearly articulated. There were rich and recurrent descriptors of the type of actions needed. *Coordinated, integrated* and *intersectoral actions* were highlighted most frequently and this was well stated by Catford and Cateson [129]: “A complex web of action is needed to address the underlying social, cultural, physical, and economic determinants … A combination of approaches is required, delivered in a coordinated way, with the input of a range of different sectors and organisations.” (p579). Coordinated, integrated planning and implementation of action was discussed as requiring a shared foundation of values, principles and accountability [27, 52, 93]. These three requirements may form a good basis for studying and understanding the lack of implementation. Furthermore, similar to the discussion about diverse entities as inherent in systems for CDP, there was a significant discussion about coordination of intersectoral action involving non-health sectors [32, 42, 62, 95, 111, 119, 123, 125, 130–132]. The key is the synergistic effects from coordinated actions [52] and system integration that could result [29].

Effective CDP actions were also discussed in terms of *multifaceted, comprehensive and knowledge-based* approaches. For example, multifaceted actions include efforts to change individual behaviour, strengthen community and environments for health, as well as the implementation of health promoting policy [45, 63, 85, 110, 133]. These are clearly aligned with the Ottawa Charter [134] strategies however this document did not surface as a foundation in the literature reviewed. Comprehensiveness was not only reported in terms of multiple strategies or actions but also in terms of comprehensive implementation of plans that include monitoring targets and responsibilities among entities [27, 93]. Furthermore, evidence informed or knowledge-based actions were discussed as a critical dimension [29, 52, 76, 89, 116, 135] and these dimensions are closely linked and discussed below with respect to information as an attribute of effective system for CDP.

In addition, actions at multiple levels were consistently reported as fundamental [17, 32, 59, 61, 71, 109, 122, 124, 126, 133, 135] and this is in keeping with the description of effective systems for CDP above. An increased focus on the complexity of implementing several strategies at multiple levels and tailored to changing contexts was reported as important [5, 74].

There was considerable attention to the leadership role of the *health care sector***,** particularly primary health care, in implementing actions for CDP [2, 32, 81, 85, 126]. Key recommendations included increasing the priority on CDP action [73, 74], targeting patient, provider, and system level factors [122, 127], improving services and community programs [68, 133], and enhancing organizational capacity in health systems [96]. Following this, the need for leadership [108] and particularly increased primary health care coordination of programs or services within health departments was deemed necessary [37, 68, 113, 133, 135]. There was, however, a tendency in the literature to retreat to discussion of chronic disease management [106, 133].

Lastly, some literature focused on the relationships *among diverse actions* [51, 68, 70, 89, 109, 122]. Gortmaker et al. [27] reported that addressing these types of relationships requires complex systems thinking to conceptualise systemic causes and to organise evidence needed to plan and implement action. Further, a systems approach was described as addressing the relationships among multiple, heterogeneous interdependent factors (i.e., actors, entities and actions) that interact dynamically across levels of action [17, 55, 136]. Overall, the connections among attributes such as collaborative capacity, leadership, information and implementation of action were clearly evident.

#### Information

Three themes are combined to form information as an attribute of effective systems for CDP: surveillance of chronic diseases and monitoring of system performance, research and evaluation, and knowledge exchange. Some literature, however, discussed information more broadly in terms of multicomponent monitoring systems examining policy, environments, health system responses, capacity building, innovation, and accountability [16, 71, 104].

Sensitive and context- specific surveillance of the epidemiology of chronic disease was deemed vital to CDP [14, 15, 54, 55, 113, 121]. There was ample discussion of the need to embed surveillance in CDP goals and to ensure accountability mechanisms are in place [14, 15, 33, 39, 94, 121, 130, 132, 133]. Similar to calls for broad monitoring systems, Baum et al. discussed the need for embedded health equity goals that require the production of relevant data and evidence not simply the monitoring of chronic disease. Thus, connections became clear as to the links between surveillance and monitoring, leadership, governance and accountability, and health equity paradigms. Overall, building accountability mechanisms and capacity to monitor progress towards goals and measureable targets were emphasized [67, 68, 73, 85, 117, 121, 123, 131, 132].

There was considerable discussion of research and evaluation in effective systems for CDP. A key theme was the importance of building research capacity [14, 27, 54, 65, 66, 76, 77] and as Huang et al. [17] pointed out this requires multilevel research involving leadership among researchers and policy makers, public-private sectors, and national governments. Ali et al. [15] emphasized the participation of decision- makers in research and Manson et al. [14] indicated linking research with political agendas to be vital. Some literature indicated that participatory action research methods are aligned well with complex systems approaches particularly with respect to bringing diverse perspectives together to address the complexity of CDP [52, 75]. Thus, the interdependence of collaborative capacity, leadership and research in effective systems for CDP was recognized.

The increased need for evaluation of implemented CDP actions was commonly identified in the literature. This was discussed in terms of demonstrated commitment to and funding for evaluation of policies and programs [27, 42, 54, 55, 93] and linking these to quality improvement [89]. One area not commonly reported was economic evaluation. As Gortmaker [27] stated, **“**a systems approach reminds us of the importance of structural or cross-cutting interventions that support direct actions, but for which cost-effectiveness evidence is not available” (p842)**.**

There was some discussion of the challenges to evaluation from a complex systems perspective for example, accounting for the complexity of urban planning [93], measuring outcomes of CDP networks [72] and evaluating the impact of advocacy activities [109]. In keeping with how systems for CDP are conceptualized in this paper, Matheson et al. (2017) highlight that evidence is context specific therefore factors such as history, resources, and organization of the system needs to be examined and considering different perspectives on the criteria that should be applied in evaluation is part of understanding complex systems.

Finally, structures and processes for knowledge exchange or the translation of existing research evidence to purposeful action for diverse audiences (i.e., public, health providers, and policy makers) was commonly identified as critical to effective systems for CDP [42, 49, 52, 57, 59, 60, 65, 75, 89, 93, 109, 115]. Wutzke et al. [74] described this in broad terms including increasing demand for prevention through improved communication about effective systems for CDP, learning from global and national evidence regarding best practices and ensuring feedback-learning cycles are supported.

#### Resources

Much of the literature utilized *resources* as a catchall term, for example, Bloch et al. [52] listed material, financial, time, expertise, and creative thinking as vital resources for CDP. Supportive infrastructure was another term used to encapsulate diverse resources [55]. These conceptualizations are different from the health system building block framework [6] where financing and workforce are discrete building blocks.

Resource development figured prominently in terms of the need to mobilize core, adequate or appropriate resources and long-term investment [27, 32, 37, 42, 54, 65, 80, 101, 129, 137]. Beyond discussing resources for the implementation of desired actions and research [74] resources to build collaborative capacity were called for to facilitate synergistic effects of linked actions [30, 82], shared or leveraged cross sectoral assets [42, 59, 89] and system integration and coordination [29].

With respect to financial resources, the literature clearly emphasized that stable, sustained and flexible funding was required to support CDP [5, 14, 15, 82]. Similar emphasis with respect to human resources was found and there was also considerable discussion of the need for workforce development. For example, skill and competency development among practitioners was advocated [14, 40, 137] in areas such as health in all policies [101, 107], working in multidisciplinary teams [10, 51, 59, 105, 138] and cross-sectoral collaboration [15, 75, 80, 105, 107, 124]. Finally, technological resources for CDP were discussed to a very limited extent. When specified as important these resources were discussed in terms of technology to aggregate and use information [60, 89] and to enable communication [81].

#### Complex systems paradigm

As per the study design, having an overarching complex systems perspective or paradigm was identified as an attribute of effective systems for CDP in some literature [74, 139]. This perspective was viewed as holistic, ecological or whole system thinking [52, 139] that required a paradigm shift [5, 75] to address the complexity of systems for CDP [55, 136, 140]. A complex systems paradigm was also described in terms of strategic [139], intelligent and high level system design [15, 120, 139] to help identify “enablers, accelerants, synergies, and interconnectedness of multiple influences” [76] and facilitate context sensitive and cross cutting actions and strategies to strengthen systems for CDP [52]. Further, complex systems perspectives were reported to be complimentary to socioecological models but they specifically aim to advance understanding of dynamic interaction among such things as the heterogeneity and relationships among individuals, entities and subsystems, nonlinear and overlapping interdependencies among entities and actions at multiple levels, and feedback mechanisms and delays [136].

Huang et al. [17] stated that “faced with the continued lack of effective and sustainable prevention strategies” systems perspectives are needed to address issues such as obesity (p7). Calls for systems approaches were linked with calls for increased attention to the implementation of actions, for example, Gortmaker et al. [27] stated that complex systems thinking is useful to conceptualise and organise evidence needed for implementing CDP action. Further to this, Wutzke et al. [5, 74] explained that a systems perspective shifts thinking regarding traditional prevention strategies because the focal points are the numerous actors, paradigms, processes, actions, contexts, structures and the dynamic interactions between them that either facilitate or hinder CDP.

Some complex systems thinking methods and tools were described in the selected literature however there was no synthesis nor consensus. System mapping was discussed most frequently and there were several different approaches reported. For example, Maclean et al. [141] mapped system entities at various levels and key influences to CDP in a flow chart type diagram while Bloch et al. [52] and Willis et al. [83] created word and arrow type diagrams to illustrate the interactions of factors that influenced the system for CDP. There was attention to system mapping to assess various attributes of systems (some discussed in this paper) and identify leverage points to strengthen systems change. Assessing and building collaborative capacity among diverse entities through system mapping was the most described approach. For example, Contandriopoulos [24] contended that “chronic disease prevention should not be conceived as the sum of discrete CDP-active organizations’ capacities, but rather as a more complex ecology of interconnected organizations whose overall influence is shaped by the ways in which they are interconnected and collaborate” (p112). Other literature discussed system mapping in order to support the identification of structures and processes to build participation and group learning about context, synergies, interactions and collaboration [15, 30, 33, 52, 75, 139]. System mapping was also used to assess resources, for example, determine diverse and valuable resources [52], identify strategic and well connected organizations as “vectors” to transfer resources [24], leverage cross sectoral assets [89] and understand human resources in terms of roles in diverse entities [15]**.** Finally, systems mapping was used for identifying the infrastructure to build effective knowledge exchange processes and structures, enhance cultures of learning, and highlight potential measures for monitoring and accountability [24, 30].

Rating and comparing various attributes of systems for CDP was also a method reported in the selected literature. Martin et al. [25] used a benchmarking tool to rate nine domains or desirable actions (e.g., regulations, policies) to address obesity and presented findings in a graphical radar chart. Other examples included the use of tables to convey ratings of domains of organizational capacity domains [47], a healthy city [19], and of monitoring accountability systems for CDP [71].

Further to these approaches, there were examples of causal loop diagrams to map or illustrate feedback in systems. Waqa et al. [139] described these diagrams as a “grounded logic model of the complex drivers that help to illustrate interdependent cause-effect relationships that either facilitate or hinder the underlying infrastructure of the system” (p2). Here, the causal loop diagram was a product of framing the dynamic problem, creating connection circles, and group model building using systems dynamics approaches. This system dynamics tool brought a complexity-focused lens to system mapping that went beyond the linear word and arrow diagrams.

Concept mapping as a method was described in two articles. Wutzke et al. [74] contended that this method (i.e., qualitative brainstorming and quantitative cluster analysis) was useful for gathering diverse perspectives on the most important and feasible multi-stakeholder and multi-component CDP actions. Concept mapping was also used by Manafo et al. [33] and they too identified many actions across numerous clusters and took these forward to consultation workshops to determine collaborative opportunities and strategic investment in actions.

Only Contandriopoulos et al. [24] discussed the application of social network analysis. They used this method “to map interorganizational collaborative networks”, “measure the structural properties of the network” and “present visually the patterns of connections used for sharing resources and information” (p.e110). Thus, this method appeared to be useful for assessing not only potential collaborative capacity but also other attributes such as resources and information.

Document review was used to analyze policies that addressed obesity through the lens of a systems-based intervention-level framework (i.e., paradigm, goals, system structure, feedback and delays and structural elements) [136]. The framework was considered valuable to identify interventions “that range from targeting specific groups of people and a specific behavior to affecting the deeply held beliefs that underlie the actions of actors throughout the system” (p. 1277). This framework was useful to assess CDP actions in terms of the degree to which they could potentially leverage system change.

Finally, although computational or quantitative modelling was recommended there were no applications discussed in the selected literature. For example, Huang et al. [17] and Gortmaker et al. [27] simply called for studies of this type and Lowe et al. [93] explained the usefulness in terms of “simplifying reality into a conceptual model, which can then be used to predict the potential effects of a policy or plan on a range of inter-related health risk factors” (p.16).

## Discussion

**Fig. 2:**
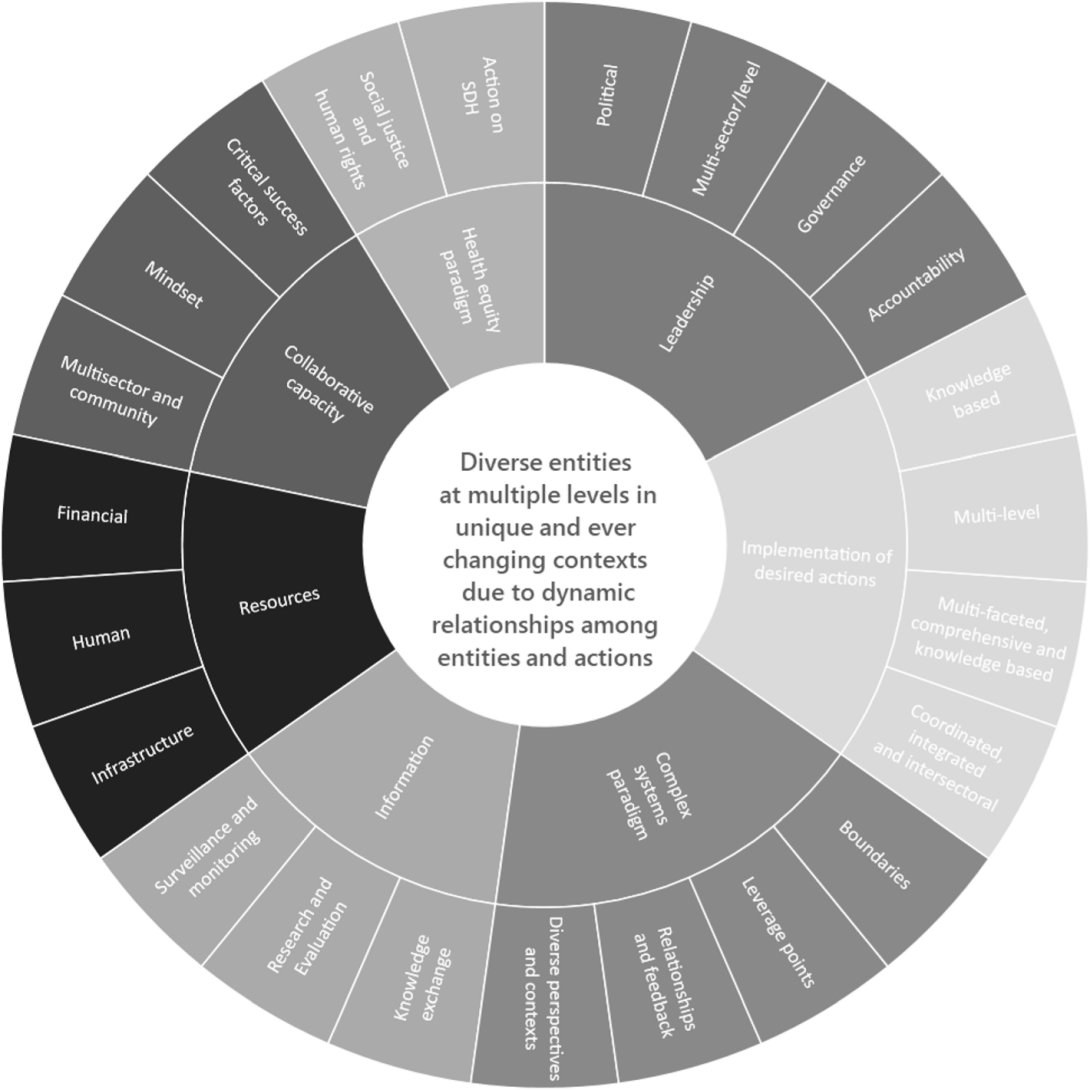
Framework for describing, assessing and strengthening systems for CDP

The aim of this paper is to provide a foundation for the development of a practical framework to strengthen systems for CDP. By building upon the literature in a systematic manner and answering our research questions, we drafted a description of systems for CDP and identified seven attributes (and dimensions) of effective systems for CDP. These findings were used to create a framework illustrated in Fig. 2. In the following section we discuss key aspects of the framework, preliminary implications for practical application and directions for future research.

First, the description of a system for CDP is placed at the centre of the framework (Fig. 2). The description is not particularly novel (i.e., diverse entities and actions), however, the embedded complex systems perspective is a good contribution. This sets up the emerging significance of a complex systems paradigm as a key attribute and the associated dimensions of dynamic interactions among diverse perspectives and contexts, relationships and feedback, boundaries of systems and leverage points to strengthen systems for CDP.

One practical implication of having a description of systems for CDP at the centre of the framework is to consider mapping key system entities and actors. This builds on the literature and would be helpful to clearly identify boundaries or what and who are in and out of the system under study [12]. There was little in the selected literature that explains how to create systems maps, however, there are several resources to help draw “a visual depiction of the key organizations and/or individuals that make up a system, including those directly affected by the system as well as those whose actions influence the system” [142]. Overall, system maps can help focus attention on the parts as well as the whole system and focus on attributes such as collaborative capacity (Fig. 2).

However, the selected literature was not strong in terms of what Ulrich [143] labels boundary critique. This is a critical systems thinking approach to assess which facts and values influence selectivity in determining boundaries of any system. Boundary critique points to critical reflection on how the systems for CDP may be described and assessed with respect to

- whose interests are/should be served in strengthening the system change and what might the consequences be?
- who are/ought be the decision makers in strengthening systems and what resources and measures of success do they have control over?
- who is/should be involved as provider of evidence and experience? and
- who is/should be considered legitimate stakeholders or actors and what diverse perspectives or worldviews are/should be considered? [143, 144].

Boundary critique illuminates issues of power and would be particularly instrumental for describing and assessing systems for CDP where public-private sector partnerships are in the fore.

Following this, although contextual factors were emphasized as critical to describing effective systems for CDP there was little in the selected literature regarding methods and tools to capture these factors and monitor change over time. Although the need to measure system change broadly through participatory evaluation was discussed [16] as was the need for research regarding “structural aspects of the social, physical, and policy environment” [17] further examination of methods and tools to monitor the changing context of systems for CDP would be a valuable addition to the framework.

Second, the suite of attributes and their dimensions indicated in Fig. 2 are also not particularly novel, however, we believe it is the confirmation, consolidation and configuration that is new. We have not found another framework that builds on the literature and consolidates key themes in this manner. Various combinations of attributes were found and Manson et al’s [14] discussion of enabling capacities and Farrington et al’s [19] strategic attributes were most similar. However, in these examples the dynamic nature of relationships among attributes and actions were not explicitly discussed. Furthermore, few authors discussed the health system building blocks framework [54, 99, 139]. Although Waqa et al. [139] discussed the building blocks and the application of complex systems approaches it was the food system that was under study and did not address the breadth and scope of systems for CDP that was the focus of this paper. Overall, we anticipated and found that attributes such as health equity and complex systems paradigms emerged as important in systems for CDP but these were not included as discrete health system building blocks in World Health Organization framework.

To strengthen the framework a review of the CDP literature regarding the implementation and evaluation of complex interventions in complex systems [145], social innovation [146] and system leadership [147] would be valuable as this literature was not included in this paper. Additionally, a review of all attributes through a broad health promotion lens would be valuable to enhance completeness, comprehensiveness, and/or clarity of dimensions. For example, a review of the health equity paradigm [148] as a key attribute of effective systems for CDP is particularly recommended because we found little practical discussion in the selected literature. Integration of literature that synthesizes the implementation of desired actions for CDP such as those articulated by Calder et al. [149] is an obvious and critical addition to buttress the framework. Another example is to integrate the vast literature on critical success factors of multisectoral partnerships or intersectoral collaboration [30, 77, 150] and building community capacity and empowerment [151–153] as dimensions of the collaborative capacity attribute.

Third, the selected literature provided a small sample of potential complex systems methods and tools to describe, assess and strengthen systems for CDP. An expanded review of the complex systems approaches is recommended, for example, the inclusion of quantitative dynamic simulation modelling to discern policy options in CDP [154]. At the very least a review of methods and tools to assess attributes identified in this framework is needed to discover critical leverage points to strengthen systems for CDP. Thus, future research should articulate and pilot methods and tools with engaged policy makers and practitioners and areas for consideration could include:

- benchmarking the extent to which attributes are present (e.g., surveys, interviews, social network analysis),
- identifying relationships and interdependencies among attributes and contextual factors (e.g., focus groups, word and arrow diagrams, feedback loops),
- creating causal loops diagrams of interactions and feedback mechanisms (e.g., group model building), and
- pinpointing key facilitating and hindering interactions and identifying the most important and feasible leverage points to strengthen systems for CDP (e.g., concept mapping).

Finally, the purpose of this paper was to develop a practical framework to strengthen systems for CDP and therefore action, evaluation and ongoing learning are central to its application. Although there was discussion of the need for evaluation of CDP actions there was little discussion about the broad topic of evaluating systems change. Therefore, we recommend that developmental and realist evaluation perspectives would be valuable to the future development of the framework. According to Patton [155], key elements of developmental evaluation are to acknowledge complex systems and problems (as opposed to simple or complicated) and focus on process, patterns and relationships, and interpretative dialogue. Realist evaluation [156] would also be useful as it asks “what works, for whom, and under what circumstances” and is based upon identifying unique contexts, mechanisms and outcomes to answer these questions. To develop robust evaluation and monitoring of systems change, Preskill et al. [157] list several additional methods and tools that should be considered (e.g., rapid feedback debriefs, timeline of key events, reflective journals, most significant change, ripple effect mapping, appreciative inquiry, observations and surveys).

This literature review excluded the abundant literature with respect to evaluations of topic specific intervention effectiveness. For example, there is rich literature to be reviewed with respect to systems thinking and healthy eating, active living and childhood obesity [158, 159] and tobacco reduction [18]. We expect this literature would provide additional insights as to attributes of effective systems for CDP and the application of complex systems thinking in research and practice. Further, the review criteria was tightly focused on systems for CDP and literature that informs broad health promotion policy and practice [160] and structural and commercial determinants of health [39] would also provide important additions to the framework. Finally, a review of settings approaches to CDP such as workplaces [161] should be included to explore potential context specific features to the framework.

## Conclusion

The urgency to increase demand and effort to reduce chronic disease is well-documented and the results of this literature review provide a strong foundation for a framework to help strengthen systems for CDP. The framework consolidates not only well-established attributes of effective CDP but also highlights theoretical and practical insights from complex systems perspectives. Although there is much work to be done to further develop the framework it is time to engage with interested policy makers, practitioners and community members to pilot its use with promising methods and tools because systems for CDP must be strengthened if they are to effectively address what is arguably the biggest threat to population health today.

## Additional file

Additional file 1: Results of coding selected literature. This file contains the results of a coding process. Selected literature (*n* = 141) was coded in terms of the extent to which the research questions and the working definition of systems for CDP were addressed. (DOCX 32 kb)

## Data Availability

All data generated or analysed during this study are included in this published article and its supplementary information file.

## Abbreviation

CDP: Chronic disease prevention
