# Supplementary material for: Strengthening complex systems for chronic disease prevention: a systematic review
Source: BMC Public Health. 2019 Jun 11;19:729. doi: 10.1186/s12889-019-7021-9 (PMC6558784; doi:10.1186/s12889-019-7021-9)
Supplement: Supplementary file 1 — Results of coding selected literature. This file contains the results of a coding process. Selected literature (n = 141) was coded in terms of the extent to which the research questions and the working definition of systems for CDP were addressed. (DOCX 32 kb) [file 12889_2019_7021_MOESM1_ESM.docx]

Additional File 1: Results of coding literature

Literature coded to a limited extent:

1. Alberti, K. G. M. M., Bailey, C. J., Blonde, L., Felton, A. M., & Zimmet, P. (2007). Partnering with governments and other institutions: Driving change in diabetes care. *International Journal of Clinical Practice, 61*(SUPPL. 157), 38-46. doi:10.1111/j.1742-1241.2007.01615.x
2. Allen, P., Sequeira, S., Best, L., Jones, E., Baker, E. A., & Brownson, R. C. (2014). Perceived benefits and challenges of coordinated approaches to chronic disease prevention in state health departments. *Preventing Chronic Disease, 11*(5).
3. Alwan, A., & MacLean, D. R. (2009). A review of non-communicable disease in low- and middle-income countries. *International Health, 1*(1), 3-9. doi:10.1016/j.inhe.2009.02.003
4. Anderson, P., Harrison, O., Cooper, C., & Jane-Llopis, E. (2011). Incentives for health. *Journal of Health Communication, 16*(SUPPL. 2), 107-133. doi:10.1080/10810730.2011.601531
5. Arora, M., Chauhan, K., John, S., & Mukhopadhyay, A. (2011). Multi-sectoral action for addressing social determinants of noncommunicable diseases and mainstreaming health promotion in national health programmes in India. Indian journal of community medicine: official publication of Indian Association of Preventive & Social Medicine, 36(Suppl1), S43.
6. Beaglehole, R., & Yach, D. (2003). Globalisation and the prevention and control of non-communicable disease: The neglected chronic diseases of adults. *Lancet, 362*(9387), 903-908. doi:10.1016/S0140-6736(03)14335-8
7. Catford, J. C., & Caterson, I. D. (2003). Snowballing obesity: Australians will get run over if they just sit there. *Medical Journal of Australia, 179*(11), 577-579.
8. Der Ananian, C., & Ainsworth, B. (2013). Population based approaches for health promotion. *Deutsche Zeitschrift fur Sportmedizin, 64*(6), 166-169. doi:10.5960/dzsm.2012.079
9. Jarris, P. E., & Schneider, J. P. (2013). Engaging Partners to Support the National Prevention Strategy. *Journal of Public Health Management and Practice, 19*(4), 386-387.
10. Kelly, B. B., Narula, J., & Fuster, V. (2012). Recognizing global burden of cardiovascular disease and related chronic diseases. *The Mount Sinai Journal Of Medicine, New York, 79*(6), 632-640. doi:10.1002/msj.21345
11. Kent, J., & Thompson, S. (2012). Health and the built environment: Exploring foundations for a new interdisciplinary profession. *Journal of Environmental and Public Health, 2012*. doi:10.1155/2012/958175
12. Krist, A. H., Shenson, D., Woolf, S. H., Bradley, C., Liaw, W. R., Rothemich, S. F., . . . Anderson, L. A. (2013). Clinical and community delivery systems for preventive care: An integration framework. *American Journal of Preventive Medicine, 45*(4), 508-516. doi:10.1016/j.amepre.2013.06.008
13. Macfarlane, R. G., Wood, L. P., & Campbell, M. E. (2014). Healthy Toronto by Design: Promoting a healthier built environment. *Canadian Journal Of Public Health = Revue Canadienne De Sante Publique, 106*(1 Suppl 1), eS5-eS8. doi:10.17269/cjph.106.3855
14. Martin, J., Peeters, A., Honisett, S., Mavoa, H., Swinburn, B., & De Silva-Sanigorski, A. (2014). Benchmarking government action for obesity prevention - An innovative advocacy strategy. *Obesity Research and Clinical Practice, 8*(4), e388-e398. doi:10.1016/j.orcp.2013.07.001
15. Mayes, R., & Armistead, B. (2013). Chronic disease, prevention policy, and the future of public health and primary care. *Medicine, Health Care and Philosophy, 16*(4), 691-697. doi:10.1007/s11019-012-9454-0
16. Mayes, R., & Oliver, T. R. (2012). Chronic disease and the shifting focus of public health: Is prevention still a political lightweight? *Journal of Health Politics, Policy and Law, 37*(2), 181-200. doi:10.1215/03616878-1538593
17. Mays, G. P., & Scutchfield, F. D. (2010). Improving Public Health System Performance Through Multiorganizational Partnerships. *Preventing Chronic Disease, 7*(6).
18. Meisel, J. D., Sarmiento, O. L., Montes, F., Martinez, E. O., Lemoine, P. D., Valdivia, J. A., . . . Zarama, R. (2014). Network analysis of bogotá's ciclovía recreativa, a self-organized multisectorial community program to promote physical activity in a middle-income country. *American Journal of Health Promotion, 28*(5), e127-e136. doi:10.4278/ajhp.120912-QUAN-443
19. Mendis, S., & Chestnov, O. (2014). The global burden of cardiovascular diseases: a challenge to improve. *Current Cardiology Reports,* 16(5), 486-486. doi:10.1007/s11886-014-0486-3
20. Mensah, G. A., Dietz, W. H., Harris, V. B., Henson, R., Labarthe, D. R., Vinicor, F., & Wechsler, H. (2005). Prevention and control of coronary heart disease and stroke--nomenclature for prevention approaches in public health: a statement for public health practice from the Centers for Disease Control and Prevention. *American Journal of Preventive Medicine, 29*(5 Suppl 1), 152-157.
21. Politis, C. E., Mowat, D. L., & Keen, D. (2017). Pathways to policy: Lessons learned in multisectoral collaboration for physical activity and built environment policy development from the Coalitions Linking Action and Science for Prevention (CLASP) initiative. *Canadian Journal Of Public Health = Revue Canadienne De Sante Publique, 108*(2), e192-e198. doi:10.17269/cjph.108.5758
22. Puska, P. (2002). Successful prevention of non-communicable diseases: 25 year experiences with North Karelia Project in Finland. *Public Health Medicine, 4*(1), 5-7.
23. Rahim, H. F. A., Sibai, A., Khader, Y., Hwalla, N., Fadhil, I., Alsiyabi, H., . . . Husseini, A. (2014). Non-communicable diseases in the Arab world. *Lancet (London, England), 383*(9914), 356-367. doi:10.1016/S0140-6736(13)62383-1
24. Raine, K. D., Sosa Hernandez, C., Nykiforuk, C. I. J., Reed, S., Montemurro, G., Lytvyak, E., & MacLellan-Wright, M.-F. (2014). Measuring the progress of capacity building in the Alberta Policy Coalition for Cancer Prevention. *Health Promotion Practice, 15*(4), 496-505. doi:10.1177/1524839913511627
25. Rebanal, R. D., & Leman, R. (2005). Collaboration between Oregon's chronic disease programs and Medicaid to decrease smoking among Medicaid-insured Oregonians with asthma. *Preventing Chronic Disease, 2 Spec no*, A12-A12.
26. Sampalli, T., Christian, E., Edwards, L., & Ryer, A. (2015). A Chronic Disease Prevention and Management Corridor© to Supporting System-Level Transformations for Chronic Conditions. *Healthcare Quarterly (Toronto, Ont.), 18*(3), 43-48.
27. Schwartz, J. I., Guwatudde, D., Nugent, R., & Kiiza, C. M. (2014). Looking at non-communicable diseases in Uganda through a local lens: An analysis using locally derived data. *Globalization and Health, 10*(1). doi:10.1186/s12992-014-0077-5
28. Seaton, C. L., Holm, N., Bottorff, J. L., Jones-Bricker, M., Errey, S., Caperchione, C. M., . . . Healy, T. (2017). Factors That Impact the Success of Interorganizational Health Promotion Collaborations: A Scoping Review. *American Journal of Health Promotion: AJHP*, 890117117710875-890117117710875. doi:10.1177/0890117117710875
29. Tangcharoensathien, V., Srisookwatana, O., Pinprateep, P., Posayandonda, T., & Patcharacarumol, W. (2017). Multisectoral Actions for Health: Challenges and Opportunities in Complex Policy Environments. *International Journal of Health Policy and Management, 6*(7), 359-363.
30. Thompson, S., Kent, J., & Lyons, C. (2014). Building partnerships for healthy environments: research, leadership and education. *Health Promotion Journal of Australia,* 25, 202-208.
31. Walkinshaw, L. P., Mason, C., Allen, C. L., Vu, T., Nandi, P., Santiago, P. M., & Hannon, P. A. (2015). Process evaluation of a regional public health model to reduce chronic disease through policy and systems changes, Washington State, 2010-2014. *Preventing Chronic Disease, 12*, E37-E37. doi:10.5888/pcd12.140446
32. Weeramanthri, T., Hendy, S., Connors, C., Ashbridge, D., Rae, C., Dunn, M., . . . Swanson, N. (2003). The Northern Territory preventable chronic disease strategy--promoting an integrated and life course approach to chronic disease in Australia. *Australian health review : a publication of the Australian Hospital Association, 26*(3), 31-42.

Literature coded to some extent

1. Adams, O. (2015). Policy Capacity for Health Reform: Necessary but Insufficient: Comment on "Health Reform Requires Policy Capacity*". International Journal Of Health Policy And Management*, 5(1), 51-54. doi:10.15171/ijhpm.2015.164
2. Allen, L., & Bloomfield, A. (2016). Engaging the private sector to strengthen NCD prevention and control. *The Lancet Global Health*, 4(12), e897-e898.
3. Amuyunzu-Nyamongo, M., Owuor, J. O., & Blanchard, C. (2013). The Consortium for NCD Prevention and Control in Sub-Saharan Africa (CNCD-Africa): From concept to practice. *Global Health Promotion*, 20(4_suppl), 97-103. doi:10.1177/1757975913500682
4. Anderson, P., Harrison, O., Cooper, C., & Jane-Llopis, E. (2011). Incentives for health. *Journal of Health Communication*, 16(SUPPL. 2), 107-133. doi:10.1080/10810730.2011.601531
5. Barr, V. J., Robinson, S., Marin-Link, B., Underhill, L., Dotts, A., Ravensdale, D., & Salivaras, S. (2003). The expanded Chronic Care Model: an integration of concepts and strategies from population health promotion and the Chronic Care Model. *Hospital quarterly*, 7(1), 73-82.
6. Bauer, U. E., Briss, P. A., Goodman, R. A., & Bowman, B. A. (2014). Prevention of chronic disease in the 21st century: elimination of the leading preventable causes of premature death and disability in the USA. *Lancet (*London, England), 384(9937), 45-52. doi:10.1016/S0140-6736(14)60648-6
7. Baum, F., & Fisher, M. (2011). Are the national preventive health initiatives likely to reduce health inequities? (Special Issue: Rethinking Health Inequity at a Time of Reform: Theory, Policy and Practice.). *Australian Journal of Primary Health*, 17(4), 320-326.
8. Baum, F., & Simpson, S. (2006). Building healthy and equitable societies: what Australia can contribute to and learn from the Commission on Social Determinants of Health. *Health Promotion Journal of Australia,* 17(3), 174-179.
9. Baum, F. E., Bégin, M., Houweling, T. A. J., & Taylor, S. (2009). Changes not for the fainthearted: Reorienting health care systems toward health equity through action on the social determinants of health. *American Journal of Public Health*, 99(11), 1967-1974. doi:10.2105/AJPH.2008.154856
10. Beaglehole, R., Bonita, R., Alleyne, G., Horton, R., Li, L., Lincoln, P., . . . Stuckler, D. (2011). UN high-level meeting on non-communicable diseases: Addressing four questions. *The Lancet*, 378(9789), 449-455. doi:10.1016/S0140-6736(11)60879-9
11. Beaglehole, R., Bonita, R., Horton, R., Adams, C., Alleyne, G., Asaria, P., . . . Watt, J. (2011). Priority actions for the non-communicable disease crisis. *The Lancet*, 377(9775), 1438-1447. doi:10.1016/S0140-6736(11)60393-0
12. Bellew, B., Schöeppe, S., Bull, F. C., & Bauman, A. (2008). The rise and fall of Australian physical activity policy 1996 - 2006: a national review framed in an international context. *Australia And New Zealand Health Policy,* 5, 18-18. doi:10.1186/1743-8462-5-18
13. Bonita, R., Magnusson, R., Bovet, P., Zhao, D., Malta, D. C., Geneau, R., . . . Beaglehole, R. (2013). Country actions to meet UN commitments on non-communicable diseases: A stepwise approach. *The Lancet*, 381(9866), 575-584. doi:10.1016/S0140-6736(12)61993-X
14. Bull, F. C., Bellew, B., Schöppe, S., & Bauman, A. E. (2004). Developments in National Physical Activity Policy: an international review and recommendations towards better practice. *Journal Of Science And Medicine In Sport*, 7(1 Suppl), 93-104.
15. Buse, K., Tanaka, S., & Hawkes, S. (2017). Healthy people and healthy profits? Elaborating a conceptual framework for governing the commercial determinants of non-communicable diseases and identifying options for reducing risk exposure. *Globalization and Health*, 13(34).
16. Chan, J. C. N., Zhang, Y., & Ning, G. (2014). Diabetes in China: A societal solution for a personal challenge. *The Lancet Diabetes & Endocrinology*, 2(12), 969-979. doi:10.1016/S2213-8587(14)70144-5
17. Clark, J. (2014). Medicalization of global health 3: The medicalization of the non-communicable diseases agenda. *Global Health Action*, 7(SUPP.1). doi:10.3402/gha.v7.24002
18. Corbett, S. J. (2005). A ministry for the Public's Health: An imperative for disease prevention in the 21st century? *Medical Journal of Australia*, 183(5), 254-257.
19. Dain, K. (2017). Challenges Facing Global Health Networks: The NCD Alliance Experience: Comment on “Four challenges that global health networks face*". International Journal of Health Policy and Management*, 6(x), 1-4.
20. Davies, S. C., Winpenny, E., Ball, S., Fowler, T., Rubin, J., & Nolte, E. (2014). For debate: A new wave in public health improvement. *The Lancet*, 384(9957), 1889-1895. doi:10.1016/S0140-6736(13)62341-7
21. De Jager, P., Hofman, K., Khan, T., Volmink, H., & Jina, R. (2012). Issues in medicine recommendations to improve the national development plan for health. *South African Medical Journal*, 102(11), 827-829. doi:10.7196/SAMJ.6160
22. de-Graft Aikins, A., Boynton, P., & Atanga, L. L. (2010). Developing effective chronic disease interventions in Africa: insights from Ghana and Cameroon. *Globalization and Health*, 6(6).
23. Demaio, A. R., Nielsen, K. K., Tersbol, B. P., Kallestrup, P., & Meyrowitsch, D. W. (2014). Primary Health Care: a strategic framework for the prevention and control of chronic non-communicable disease. *Global Health Action*, 7(24504).
24. DeSalvo, K. B., Wang, Y. C., Harris, A., Auerbach, J., Koo, D., & O'Carroll, P. (2017). Public Health 3.0: A Call to Action for Public Health to Meet the Challenges of the 21st Century. *Preventing Chronic Disease*, 14.
25. Duran, A., & Khot, A. (2011). Strengthening the health system to better confront noncommunicable diseases in India. *Indian Journal of Community Medicine*, 36(SUPPL.), S32-S37. doi:10.4103/0970-0218.94706
26. Farrington, J. L., Faskunger, J., & Mackiewicz, K. (2015). Evaluation of risk factor reduction in a European City Network*. Health Promotion International*, 30 Suppl 1, i86-i98. doi:10.1093/heapro/dav038
27. Fazli, G. S., Creatore, M. I., Matheson, F. I., Guilcher, S., Kaufman-Shriqui, V., Manson, H., . . . Booth, G. L. (2017). Identifying mechanisms for facilitating knowledge to action strategies targeting the built environment*. BMC Public Health,* 17(1), 1-1. doi:10.1186/s12889-016-3954-4
28. Friel, S., Harris, P., Simpson, S., Bhushan, A., & Baer, B. (2015). Health in All Policies Approaches: Pearls from the Western Pacific Region. *Asia and the Pacific Policy Studies*, 2(2), 324-337. doi:10.1002/app5.89
29. Giles-Corti, B., Vernez-Moudon, A., Reis, R., Turrell, G., Dannenberg, A. L., Badland, H., . . . Owen, N. (2016). City planning and population health: a global challenge. *The Lancet,* 388(10062), 2912-2924. doi:10.1016/S0140-6736(16)30066-6
30. Goenka, S., Prabhakaran, D., Ajay, V. S., & Reddy, K. S. (2009). Preventing cardiovascular disease in India-translating evidence to action. *Current Science*, 97(3), 367-377.
31. Gostin, L. O., Abou-Taleb, H., Roache, S. A., & Alwan, A. (2017). Legal priorities for prevention of non-communicable diseases: innovations from WHO's Eastern Mediterranean region. *Public Health*, 144, 4-12. doi:10.1016/j.puhe.2016.11.001
32. Greenberg, H., Raymond, S. U., & Leeder, S. (2011). The Prevention of Global Chronic Disease: Academic Public Health's New Frontier. *American Journal of Public Health*, 101(8), 1386-1391.
33. Hall, M., Graffunder, C., & Metzler, M. (2016). Policy approaches to advancing health equity. *Journal of* *Public Health Management and Practice*, 22, S50-S59. doi:10.1097/PHH.0000000000000365
34. Halpin, H. A., Morales-Suárez-Varela, M. M., & Martin-Moreno, J. M. (2010). Chronic disease prevention and the new public health. *Public Health Reviews*, 32(1), 120-154.
35. Henry, F. J. (2011). Obesity prevention: the key to non-communicable disease control. *The West Indian Medical Journal*, 60(4), 446-451.
36. Holman, D. M., Grossman, M., Jane Henley, S., Peipins, L. A., Tison, L., & White, M. C. (2014). Opportunities for cancer prevention during midlife: Highlights from a meeting of experts. *American Journal of Preventive Medicine*, 46(3 SUPPL. 1), S73-S80. doi:10.1016/j.amepre.2013.10.030
37. Hospedales, C. J., Samuels, T. A., Cummings, R., Gollop, G., & Greene, E. (2011). Raising the priority of chronic noncommunicable diseases in the Caribbean. *Revista Panamericana De Salud Publica = Pan American Journal Of Public* Health, 30(4), 393-400.
38. Karwalajtys, T., & Kaczorowski, J. (2010). An integrated approach to preventing cardiovascular disease: Community-based approaches, health system initiatives, and public health policy. *Risk Management and Healthcare Policy*, 3, 39-48. doi:10.2147/RMHP.S7528
39. Khandelwal, S., & Reddy, K. S. (2013). Eliciting a policy response for the rising epidemic of overweight-obesity in India. *Obesity Reviews*, 14(S2), 114-125. doi:10.1111/obr.12097
40. Khayatzadeh-Mahani, A., Ruckert, A., & Labonté, R. (2018). Obesity prevention: co-framing for intersectoral ‘buy-in’. *Critical Public Health*, 28(1), 4-11. doi:10.1080/09581596.2017.1282604
41. Kickbusch, I., Williams, C., & Lawless, A. (2014). Making the most of open windows: Establishing health in all policies in south Australia. *International Journal of Health Services*, 44(1), 185-194. doi:10.2190/HS.44.1.k
42. King, L., Turnour, C., & Wise, M. (2007). Analysing NSW state policy for child obesity prevention: strategic policy versus practical action. *Australia and New Zealand Health Policy*, 4(22).
43. King, R. J., Garrett, N., Kriseman, J., Crum, M., Rafalski, E. M., Sweat, D., . . . Cutts, T. (2016). A Community Health Record: Improving Health Through Multisector Collaboration, Information Sharing, and Technology. *Preventing Chronic Disease*, 13, E122-E122. doi:10.5888/pcd13.160101
44. Koelen, M. A., Vaandrager, L., & Wagemakers, A. (2009). What is needed for coordinated action for health? *Family Practice*, 25(SUPPL. 1), i25-i31. doi:10.1093/fampra/cmn073
45. Kottke, T. E., Stiefel, M., & Pronk, N. P. (2016). "Well-being in all policies": Promoting cross-sectoral collaboration to improve people's lives. *Preventing Chronic Disease*, 13(4). doi:10.5888/pcd13.160155
46. Kranzler, Y., Davidovich, N., Fleischman, Y., Grotto, I., Moran, D. S., & Weinstein, R. (2013). A health in all policies approach to promote active, healthy lifestyle in Israel. *Israel Journal of Health Policy Research*, 2(1). doi:10.1186/2045-4015-2-16
47. Kreindler, S. A. (2009). Lifting the burden of chronic disease: what has worked? what hasn't? what's next? *Healthcare quarterly* (Toronto, Ont.), 12(2), 30-40.
48. Labonte, R., Mohindra, K. S., & Lencucha, R. (2011). Framing international trade and chronic disease. *Globalization and Health*, 7(21).
49. Lencucha, R., Drope, J., & Chavez, J. J. (2015). Whole-of-government approaches to NCDs: the case of the Philippines Interagency Committee-Tobacco. *Health Policy And Planning*, 30(7), 844-852. doi:10.1093/heapol/czu085
50. Libman, K., Freudenberg, N., Sanders, D., Puoane, T., & Tsolekile, L. (2015). The role of urban food policy in preventing diet-related non-communicable diseases in CapeTown and New York. *Public Health*, 129(4), 327-335. doi:10.1016/j.puhe.2014.12.007
51. Liburd, L., Ehlinger, E., Liao, Y., & Lichtveld, M. (2016). Strengthening the Science and Practice of Health Equity in Public Health. *Journal of Public Health Management and Practice*, 22, S1-S4.
52. Magnusson, R. S. (2008). What's law got to do with it part 1: A framework for obesity prevention. *Australia and New Zealand Health Policy*, 5. doi:10.1186/1743-8462-5-10
53. Mamudu, H. M., Yang, J. S., & Novotny, T. E. (2011). UN resolution on the prevention and control of non-communicable diseases: An opportunity for global action. *Global Public Health*, 6(4), 347-353. doi:10.1080/17441692.2011.574230
54. Manson, H., Sullivan, T., Ha, P., Navarro, C., & Martín-Moreno, J. M. (2013). Goals are not enough: Building public sector capacity for chronic disease prevention. *Public Health Reviews*, 35(1).
55. Mariner, W. K. (2016). Beyond lifestyle: Governing the social determinants of health. *American Journal of Law and Medicine*, 42(2-3), 284-309. doi:10.1177/0098858816658268
56. McKee, M., Haines, A., Ebrahim, S., Lamptey, P., Barreto, L., Matheson, D., . . . Pearce, N. (2014). Towards a comprehensive global approach to prevention and control of NCDs. *Globalization and Health*, 10(1). doi:10.1186/s12992-014-0074-8
57. Mendis, S. (2010). The policy agenda for prevention and control of non-communicable diseases. *British Medical Bulletin,* 96, 23-43. doi:10.1093/bmb/ldq037
58. Mendis, S., & Chestnov, O. (2013). Policy reform to realize the commitments of the Political Declaration on noncommunicable diseases. *British Medical Bulletin*, 105(1), 7-27. doi:10.1093/bmb/ldt001
59. Mendis, S., & Fuster, V. (2009). National policies and strategies for noncommunicable diseases. *Nature Reviews. Cardiology,* 6(11), 723-727. doi:10.1038/nrcardio.2009.171
60. Mensah, G. A. (2003). A heart-healthy and "stroke-free" world through policy development, systems change, and environmental supports: A 2020 vision for sub-Saharan Africa. *Ethnicity and Disease*, 13(2 SUPPL. 2), S2-4-S2-12.
61. Moodie, R., Stuckler, D., Monteiro, C., Sheron, N., Neal, B., Thamarangsi, T., . . . Casswell, S. (2013). Profits and pandemics: prevention of harmful effects of tobacco, alcohol, and ultra-processed food and drink industries. *Lancet* (London, England), 381(9867), 670-679. doi:10.1016/S0140-6736(12)62089-3
62. Murnaghan, D., Morrison, W., Griffith, E. J., Bell, B. L., Duffley, L. A., McGarry, K., & Manske, S. (2013). Knowledge exchange systems for youth health and chronic disease prevention: a tri-provincial case study. *Chronic Diseases And Injuries In Canada*, 33(4), 257-266.
63. Navarro, A. M., Voetsch, K. P., Liburd, L. C., Giles, H. W., & Collins, J. L. (2007). Charting the future of community health promotion: recommendations from the National Expert Panel on Community Health Promotion. *Preventing Chronic Disease,* 4(3), A68-A68.
64. Nishtar, S., & Ralston, J. (2013). Can human resources for health in the context of noncommunicable disease control be a lever for health system changes? Bulletin of the World Health Organization, 91(11), 895-896. doi:10.2471/BLT.13.118711
65. ugent, R. (2004). Food and agriculture policy: Issues related to prevention of noncommunicable diseases. *Food and Nutrition Bulletin,* 25(2), 200-207.
66. Oldenburg, B. F., & Harper, T. A. (2008). Investing in the future: prevention a priority at last. *Medical Journal of Australia*, 189(5), 267-268.
67. Rani, M., Nusrat, S., & Hawken, L. H. (2012). A qualitative study of governance of evolving response to non-communicable diseases in low-and middle- income countries: Current status, risks and options. *BMC Public Health*, 12(1). doi:10.1186/1471-2458-12-877
68. Robinson, K., Elliott, S. J., Driedger, S. M., Eyles, J., O'Loughlin, J., Riley, B., . . . Harvey, D. (2005). Using linking systems to build capacity and enhance dissemination in heart health promotion: A Canadian multiple-case study. *Health Education Research,* 20(5), 499-513. doi:10.1093/her/cyh006
69. Robinson, K., Farmer, T., Elliott, S. J., & Eyles, J. (2007). From heart health promotion to chronic disease prevention: Contributions of the Canadian heart health initiative. *Preventing Chronic Disease*, 4(2).
70. Russell, L. M., Rubin, G. L., & Leeder, S. R. (2008). Preventive health reform: What does it mean for public health? *Medical Journal of Australia*, 188(12), 715-719.
71. Sagner, M., Arena, R., McNeil, A., Brahmam, G. N. V., Hills, A. P., De Silva, H. J., . . . Puska, P. (2016). Creating a pro-active health care system to combat chronic diseases in Sri Lanka: the central role of preventive medicine and healthy lifestyle behaviors: An Official Policy Statement of the European Society of Preventive Medicine. Expert Review of Cardiovascular Therapy, 14(10), 1107-1117. doi:10.1080/14779072.2016.1227703
72. Sagner, M., McNeil, A., Puska, P., Auffray, C., Price, N. D., Hood, L., . . . Arena, R. (2017). The P4 Health Spectrum – A Predictive, Preventive, Personalized and Participatory Continuum for Promoting Healthspan. *Progress in Cardiovascular Diseases,* 59(5), 506-521. doi:10.1016/j.pcad.2016.08.002
73. Siegel, K., & Venkat, K. M. V. (2008). The unite for diabetes campaign: Overcoming constraints to find a global policy solution. *Globalization and Health,* 4. doi:10.1186/1744-8603-4-3
74. Smith, J. A., Crawford, G., & Signal, L. (2016). The case of national health promotion policy in Australia: where to now? *Health Promotion Journal of Australia,* 27, 61-65.
75. Swinburn, B. (2008). Obesity prevention: the role of policies, laws and regulations. *Australia and New Zealand Health Policy*, 5(12).
76. Tolley, H., Snowdon, W., Wate, J., Durand, A. M., Vivili, P., McCool, J., . . . Swinburn, B. (2016). Monitoring and accountability for the Pacific response to the non-communicable diseases crisis. *BMC Public Health,* 16, 958-958. doi:10.1186/s12889-016-3614-8
77. Willis, C., Kernoghan, A., Riley, B., Popp, J., Best, A., & Milward, H. B. (2015). Outcomes of Interorganizational Networks in Canada for Chronic Disease Prevention: Insights From a Concept Mapping Study, 2015. *Preventing Chronic Disease*, 12, E199-E199. doi:10.5888/pcd12.150297
78. Wolbeck Minke, S., Smith, C., Plotnikoff, R. C., Khalema, E., & Raine, K. (2006). The evolution of integrated chronic disease prevention in Alberta, Canada. *Preventing Chronic Disease*, 3(3), A102-A102.
79. Woulfe, F., Oliver, T. R., Zahner, S. J., & Siemering, K. Q. (2010). Multisector Partnerships in Population Health Improvement. *Preventing Chronic Disease*, 7(6).
80. Wu, Y., Benjamin, E. J., & MacMahon, S. (2016). Prevention and control of cardiovascular disease in the rapidly changing economy of China. *Circulation*, 133(24), 2545-2560. doi:10.1161/CIRCULATIONAHA.115.008728
81. Yan, L. L., & Kong, L. (2016). China's Multisectoral Approach to Chronic Disease. *Global Hear*t, 11(4), 441-442. doi:10.1016/j.gheart.2016.10.026
82. Yancey, A. K., Fielding, J. E., Flores, G. R., Sallis, J. F., McCarthy, W. J., & Breslow, L. (2007). Creating a Robust Public Health Infrastructure for Physical Activity Promotion. *American Journal of Preventive Medicine,* 32(1), 68-78. doi:10.1016/j.amepre.2006.08.029

Literature coded to a great extent

1. Abernethy, P. (2016). Bridging conceptual “silos”: bringing together health promotion and sustainability governance for practitioners at the landscape scale. *Local Environment, 21*(4), 451-475. doi:10.1080/13549839.2014.968841
2. Ali, M. K., Rabadán-Diehl, C., Flanigan, J., Blanchard, C., Narayan, K. M. V., & Engelgau, M. (2013). Systems and capacity to address noncommunicable diseases in low- and middle-income countries. *Science Translational Medicine, 5*(181). doi:10.1126/scitranslmed.3005121
3. Bloch, P., Toft, U., Reinbach, H. C., Clausen, L. T., Mikkelsen, B. E., Poulsen, K., & Jensen, B. B. (2014). Revitalizing the setting approach - supersettings for sustainable impact in community health promotion. *International Journal of Behavioral Nutrition and Physical Activity, 11*(1). doi:10.1186/s12966-014-0118-8
4. Contandriopoulos, D., Hanusaik, N., Maximova, K., Paradis, G., & O'Loughlin, J. L. (2016). Mapping Collaborative Relations among Canada's Chronic Disease Prevention Organizations. *Healthcare Policy = Politiques De Sante, 12*(1), 101-115.
5. De Leeuw, E. (2017). Engagement of Sectors Other than Health in Integrated Health Governance, Policy, and Action. *Annual Review of Public Health, 38*, 329-349. doi:10.1146/annurev-publhealth-031816-044309
6. Fawcett, S., Schultz, J., Watson-Thompson, J., Fox, M., & Bremby, R. (2010). Peer reviewed: Building multisectoral partnerships for population health and health equity. Preventing chronic disease, 7(6).
7. Gortmaker, S. L., Swinburn, B. A., Levy, D., Carter, R., Mabry, P. L., Finegood, D. T., . . . Moodie, M. L. (2011). Changing the future of obesity: Science, policy, and action. *The Lancet, 378*(9793), 838-847. doi:10.1016/S0140-6736(11)60815-5
8. Harvey, P. (2005). Approaches to population health care: The emerging context! *Australian Journal of Primary Health, 11*(2), 45-52.
9. Huang, T., Drewnowski, A., Shiriki, K. A., & Glass, T. A. (2009). A Systems-Oriented Multilevel Framework for Addressing Obesity in the 21st Century. *Preventing Chronic Disease, 6*(3).
10. Johnston, L. M., Matteson, C. L., & Finegood, D. T. (2014). Systems Science and Obesity Policy: A Novel Framework for Analyzing and Rethinking Population-Level Planning. *American Journal of Public Health, 104*(7), 1270-1278.
11. Kohl III, H. W., Craig, C. L., Lambert, E. V., Inoue, S., Alkandari, J. R., Leetongin, G., . . . Wells, J. C. (2012). The pandemic of physical inactivity: Global action for public health. *The Lancet, 380*(9838), 294-305. doi:10.1016/S0140-6736(12)60898-8
12. Lobstein, T., & Brinsden, H. (2014). Symposium report: The prevention of obesity and NCDs: Challenges and opportunities for governments. *Obesity Reviews, 15*(8), 630-639. doi:10.1111/obr.12193
13. Lowe, M., Boulange, C., & Giles-Corti, B. (2014). Urban design and health: Progress to date and future challenges. *Health Promotion Journal of Australia, 25*(1), 14-18. doi:10.1071/HE13072
14. MacLean, D. R., Farquharson, J., Heath, S., Barkhouse, K., Latter, C., & Joffres, C. (2003). Building capacity for heart health promotion: Results of a 5-year experience in Nova Scotia, Canada. *American Journal of Health Promotion, 17*(3), 202-212.
15. Manafò, E., Petermann, L., Lobb, R., Keen, D., & Kerner, J. (2011). Research, practice, and policy partnerships in pan-Canadian coalitions for cancer and chronic disease prevention*. Journal of Public Health Management and Practice*, 17(6), E1-E11.
16. Magnusson, R. S., & Patterson, D. (2014). The role of law and governance reform in the global response to non-communicable diseases. *Globalization and Health, 10*(1). doi:10.1186/1744-8603-10-44
17. Matheson, A., Walton, M., Gray, R., Lindberg, K., Shanthakumar, M., Fyfe, C., . . . Borman, B. (2017). Evaluating a community-based public health intervention using a complex systems approach. *Journal Of Public* *Health* (Oxford, England), 1-8. doi:10.1093/pubmed/fdx117
18. Meyer, A. M., Davis, M., & Mays, G. P. (2012). Defining organizational capacity for public health services and systems research. *Journal of Public Health Management and Practice*, *18*(6), 535-544.
19. Rhodes, M. G. (2013). A Network Based Theory of Health Systems and Cycles of Well-being. *International Journal of Health Policy and Management, 1*(1), 7-15.
20. Robles, S. C. (2004). A public health framework for chronic disease prevention and control. (Pan American Health Organization Regional Consultation of the Americas on Diet, Physical Activity and Health.). *Food and Nutrition Bulletin, 25*(2), 194-199.
21. Satterfield, D. W., Murphy, D., Essien, J. D. K., Hosey, G., Stankus, M., Hoffman, P., . . . Alfaro-Correa, A. (2004). Using the Essential Public Health Services as strategic leverage to strengthen the public health response to diabetes. *Public Health Reports, 119*(3), 311-321. doi:10.1016/j.phr.2004.04.010
22. Waqa, G., Moodie, M., Snowdon, W., Latu, C., Coriakula, J., Allender, S., & Bell, C. (2017). Exploring the dynamics of food-related policymaking processes and evidence use in Fiji using systems thinking. *Health Research Policy and Systems, 15*(1). doi:10.1186/s12961-017-0240-6
23. Willis, C., Greene, J., & Riley, B. (2017). Understanding and improving multi-sectoral partnerships for chronic disease prevention: Blending conceptual and practical insights. *Evidence and Policy, 13*(4), 623-645. doi:10.1332/174426417X15090122455415
24. Willis, C. D., Riley, B. L., Herbert, C. P., & Best, A. (2013). Networks to strengthen health systems for chronic disease prevention. *American Journal of Public Health, 103*(11), e39-e48. doi:10.2105/AJPH.2013.301249
25. Wutzke, S., Morrice, E., Benton, M., & Wilson, A. (2016). What will it take to improve prevention of chronic diseases in Australia? A case study of two national approaches. *Australian Health Review, 41*(2), 176-181. doi:10.1071/AH16002
26. Wutzke, S., Roberts, N., Willis, C., Best, A., Wilson, A., & Trochim, W. (2017). Setting strategy for system change: Using concept mapping to prioritise national action for chronic disease prevention. *Health Research Policy and Systems, 15*(1). doi:10.1186/s12961-017-0231-7
